# Cliniciopathological Spectrum of Skin and Soft Tissue Swellings at a Tertiary Care Hospital of Lahore, Pakistan

**DOI:** 10.7759/cureus.36398

**Published:** 2023-03-20

**Authors:** Samia Fatima, Amjid ul Haq, Ali Gohar, Haseeb Mehmood Qadri, Muhammad Saad Babar, Muhammad Sheraz, Saad Abdullah, Momin Ijaz, Muhammad Awais Ahmad, Mustaqeem Rana

**Affiliations:** 1 Plastic and Reconstructive Surgery, Lahore General Hospital, Lahore, PAK; 2 Surgery, Lahore General Hospital, Lahore, PAK; 3 Surgery, Lahore General Hospital Lahore, Lahore, PAK; 4 Surgery, Jinnah Hospital, Lahore, PAK

**Keywords:** soft tissue swelling, plastic and reconstructive surgery, cosmetic dermatologic surgery, skin neurofibroma, congenital nevus, arteriovenous malformations, epidermoid cysts, giant cell tumors, cm (cutaneous melanoma) bcc (basal cell carcinoma) scc (squamous cell carcinoma)

## Abstract

Background

Skin and soft tissue swellings (SSTS) frequently present in dermatology, plastic surgery, and general surgery departments. While a general surgeon can take care of excisable lesions, people typically seek plastic surgery for cosmetic reasons. According to the signs and symptoms, soft tissue and skin lesions must be removed, and it is crucial to maintain cosmesis following their removal.

Objective

The aim of this article is to describe the clinical and histopathological types, sites, laterality, and postoperative complications of SSTS.

Material and methods

This retrospective study was conducted at the Department of Plastic and Reconstructive Surgery, Lahore General Hospital, Lahore, Pakistan in November 2022. We studied admitted patients from July 1, 2020 to June 30, 2022 for SSTS excision. Data on patients’ demographics, associated features of SSTS, and their postoperative complications was gathered using Google Docs-generated proforma and sent to a statistician for the computation of results via a Microsoft Excel-generated spreadsheet.

Results

Out of the total 60 patients, 66.7% of the lesions were found in women. The mean age at presentation came out to be 34.16±17.42 years. Nevi with 16.7% were the most common SSTS in our study. The most common site of presentation of SSTS was the scalp and face in 63.3% of cases. Fever was the most frequently encountered post-excision complication in 40% of patients.

Conclusion

A comprehensive history, clinical examination, signs and symptoms, and the histology of the lesion, all play a crucial role in the management of such swellings. Surgery was the definitive treatment option for SSTS. There were very few major complications in a handful of patients.

## Introduction

Skin and soft tissue swellings (SSTS) are frequently presented to the Department of Plastic and Reconstructive Surgery of any hospital. The origin of lesions seen in skin and soft tissue may be categorized as congenital, acquired, and infective. Based on their potential to metastasize, they can be benign or malignant lesions. A Nigerian study by Duduyemi et al. studied a similar clinicopathological spectrum of SSTS at their hospital. The most common benign skin lesions include lipoma (51%), squamous papilloma (9.79%), and dermatofibroma protuberous (9.1%). the most common malignant lesions include basal cell carcinoma (BCC, 22.73%), squamous cell carcinoma (SCC, 22.73%), and Kaposi sarcoma (13.65%) [1].

Basal cell carcinoma (BCC) is the most commonly occurring skin cancer. Basal cell carcinoma is a rarely metastasizing tumor. Other than age over 50 years, fair skin, and ultraviolet radiation (UV) exposure, immunosuppression and syndromes such as Gorlin syndrome and xeroderma pigmentosa are important risk factors in its etiopathogenesis. Hedgehog pathway activation is an important cause in the pathogenesis of BCC. Australia has the highest incidence of BCC. Two major issues reported with BCC are the destruction of the underlying dermis and recurrence. Mohs excisional surgery is the treatment of choice [2].

Cutaneous squamous cell carcinoma (cSCC) is the second most common skin cancer and the second most common form of keratinocyte carcinoma after BCC. Although many factors can increase the risk for cSCC, cumulative sun exposure, especially in childhood and youth, is of the greatest importance. In recent years, immunosuppression, including that associated with organ transplantation, has emerged as an increasingly important contributor to tumorigenesis. cSCC can develop on any skin surface [3].

Arteriovenous malformations (AVMs) account for 4.7% to 15% of all vascular anomalies, and 60% of them are congenital [4]. AVMs can occur all over the body, but the scalp accounts for 8% of them. Associated complications may include pain, bleeding, and tissue destruction [4]. Doppler ultrasound and magnetic resonance imaging (MRI) are required modalities for its accurate diagnosis. Complete surgical excision is the treatment of choice [5].

Neurofibromatosis type 1 is a multisystem developmental disorder involving the skin, central nervous system, and musculoskeletal organs forming multiple benign tumors known as neurofibromas and has an incidence ratio of 1:3000. MRI is the investigation of choice. Surgical excision and laser are the treatment options [6].

Giant cell tumor (GCT) is a rare soft tissue, radiosensitive tumor [7]. No risk factors have been identified yet. Diagnosis is based on histopathology and immunohistochemistry, involving actin and CD68. Surgical excision and radiotherapy are the treatment options [7].

Congenital melanocytic nevi (CMN) caused by mutations in embryonic precursor cells of melanocytes have an incidence of 1:100. MRI is the modality of choice for diagnosis, and surgical excision is the mainstay treatment [8].

Epidermoid cyst (EC) is a type of cutaneous cyst having an epidermal lining that produces keratin affecting male adults predominantly [9]. Risk factors include positive family history and syndromes such as Gardner’s and Gorlin’s syndromes. Surgical excision and carbon dioxide (CO2) and yttrium aluminum garnet (YAG) lasers are preferred modes of management [9].

After a careful study of the literature via PubMed Central, Google Scholar, and Scopus, we conclude that this is the first scientific study from Pakistan showcasing the clinical and pathological features of skin and soft tissue swellings presenting to the Department of Plastic and Reconstructive Surgery at a single center.

## Materials and methods

This study was conducted at the Department of Plastic and Reconstructive Surgery, Lahore General Hospital, Lahore after departmental approval from the same department, Reference # 130, dated, November 4, 2022. No human beings were involved directly in this study. It was a retrospective, observational study done in November 2022 which incorporated data of patients admitted during the past two years, i.e., from July 1, 2020 to June 30, 2022.

After a comprehensive search and study of existing English scientific literature, a pro forma was generated on Google Docs to obtain information on patient’s demographics, clinical features, site, laterality, procedure, and postoperative complications of SSTS from patients' records. All patients aged 14 years and above were included in this study, irrespective of their gender and comorbidities, who presented to us with SSTS and wanted a surgical procedure done for cosmetic reasons. Records of 63 patients were checked, out of which 60 were complete in all aspects of complying with the requirements of our pro forma. Hence, 60 is the sample size of our study.

Data compiled over a Microsoft Excel spreadsheet was sent to a statistician for analysis and compiled results were received.

## Results

**Figure 1:**
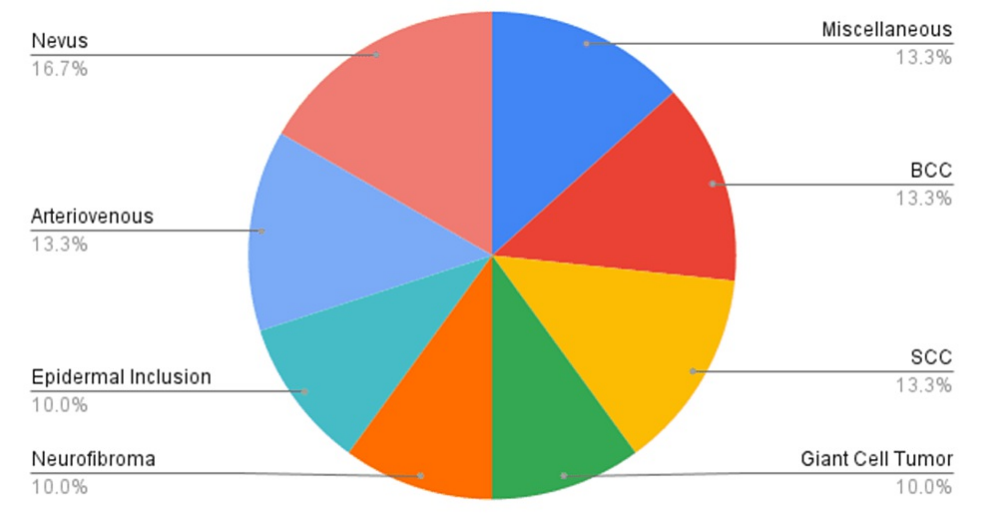
Pie chart showing types of skin and soft tissue swellings (SSTS). BCC: basal cell carcinoma; SCC: squamous cell carcinoma

A total of 60 patients were included in the study, comprising 33.3% male and 66.7% female patients. The mean age at presentation was 34.16±17.42 years. The average hospital stay was 11.5 days. The urban-to-rural ratio was 1:14. The most common type of lesion was nevus, accounting for 16.7% of the total (Figure 1). 

About 63.3% of the lesions, mostly nevi, AVMs, and BCC, were found on the face and scalp. However, SCC and GCT had a predisposition for the upper limbs. Most nevi were present on the right side, whereas arteriovenous malformations and epidermal inclusion cysts involved the midline. BCC was a left-sided lesion in the majority of the cases. The most common histopathologic type of nevus was melanocytic nevus, and BCC presented mostly as basaloid neoplasm (Table 1).

**Table 1:** Clinical features of skin and soft tissue swellings (SSTS).

Sr. #	Provisional Diagnosis	No. of Cases	Common Site	Common Side	Common Histopathologic Type
1.	Nevus	10	Face & Scalp (80%)	Right	Melanocytic nevus
2.	Arteriovenous Malformation	8	Face & Scalp (75%)	Midline	Venous malformation
3.	Basal Cell Carcinoma	8	Face (100%)	Left	Basaloid neoplasm
4.	Squamous Cell Carcinoma	8	Upper Limbs (50%), Face & Scalp (50%)	Equivocally bilateral	Squamous cell carcinoma
5.	Epidermal Inclusion Cyst	6	Scalp (100%)	Midline	Epidermal inclusion cyst
6.	Giant Cell Tumor	6	Upper Limbs (100%)	Both	Giant cell tumor
7.	Neurofibroma	6	Face & Scalp (66.7%)	Both	Neurofibroma
8.	Miscellaneous	8	Lower Limbs (50%)	Left	Spindle cell neoplasm, Glomus tumor, Dermatofibroma, Juvenile hyaline fibromatosis

The most common post-excision complication was fever, occurring in 40% of patients (Table 2).

**Table 2:** Post-excision complications of skin and soft tissue swellings (SSTS).

Sr. #	Complications	Percentage Occurrence
1.	Fever	40%
2.	Surgical Site Infection	10%
3.	Graft/flap failure	6.67%
4.	Hematoma formation	6.67%
5.	Abnormal Wound Healing	3.33%

## Discussion

This study provides an overview of the common skin and soft tissue lesions presented at our setup during a two-year period and its comparison with the existing English scientific literature.

Our study comprised 33.3% male and 66.7% female population indicating more females than males attended the hospital for SSTS surgeries, similar to the study conducted by Duduyemi et al., which revealed that 55.43% of their patients were female [1]. These results are also in line with those of a Chinese study of 2017 having a greater number of females than males [10]. Such findings suggest that females are more conscious of their cosmetic appearance and have a readily available facility of healthcare, in contrast to males who are busy with work and attend healthcare facilities in cases of extreme emergencies [1].

The mean age of presentation in our study was 34.16±17.42 years, similar to the findings of Duduyemi et al., who reported a mean age of 33.52±15.05 years, and a Chinese study in which the mean age of all the patients was 33.5 years [1, 10]. We deduce that SSTS are prevalent in young patients in their reproductive ages. The most common neoplastic and non-inflammatory lesions in this study were nevus (16.7%) followed by AVM, BCC, and SCC, each with 13.3% incidence. The results of this research are consistent with a study conducted by Jie Pan and colleagues in which the most frequent pathological types were nevi (48.96%) and epidermal inclusion cyst (EIC) (11.2%) [10].

Among nevi, melanocytic nevus was the most common histological type. Eighty percent were found on the face and scalp, with right-sided laterality in the majority of cases. A large retrospective study by Liu et al. conducted in China studied 4,561 patients with nevi of different types, including melanocytic nevi, blue nevi, halo nevi, Spitz nevi, and dysplastic nevi. They also concluded that the head and neck was the most common site of development of nevi among 66.24% of patients, with females affected in 68.01% of cases in total. About 82% of patients had melanocytic nevi, similar to the results of our study [11].

Our results suggest an incidence of 13.3% for AVM, with the face and scalp as the common site of occurrence in 75% of cases and the involvement of the upper limbs in the remaining ones. Most of them were present along the mid-axis of the head. Mathew et al. demonstrate extremities as the most common site of incidence of AVM in 53.8% of patients. AVMs were closely associated with genetic disorders, like capillary malformation AVM syndrome, Parks Weber syndrome, and hereditary hemorrhagic telangiectasia in 46.2% of patients. Although, our study had patients with isolated presentations of AVM as well [4].

The terms “sebaceous cyst” and “dermoid cyst” are used interchangeably with “epidermal inclusion cyst” (EIC), but both are actually misnomers. EICs lack sebaceous glands. Dermoid and epidermoid cysts both have keratin or cholesterol present in their cystic cavities. However, dermoid cysts have mural dermal appendages, but the latter do not have such appendages. A review article by V.T. Hoang et al. states that they are most prevalent in the third to fourth decades of life, with 7% of them affecting the head and neck region. EIC was found in 10% of patients presenting for aesthetic surgery at our department. All of them were present in the midline of the scalp [9].

Generally considered benign, giant cell tumors (GCT) are most common in the upper limbs in all age groups with no sex specificity. However, Hafiz et al. reviewed to deduce that males were affected more (58.3%) than female patients (41.7%). They have the ability to recur and acquire malignant features secondarily [7]. Likewise, all cases of GCT occurred in the upper limbs, without specific laterality in our clinical article. Six patients underwent complete surgical removal with clear surgical margins and postoperative radiotherapy to eliminate any possible chances of local recurrence.

Cannon et al. found that neurofibromas commonly involve the back, abdomen, and thigh/upper arm; however, in our study, 66.7% of lesions involved the face and scalp and 33.3% involved the lower limbs. In Cannon et al.’s study, females were affected more than males and mostly patients were in the third and fourth decades of their life, similar to our study. The feminine preponderance can be explained due to the development of neurofibromas following periods of growth in puberty and pregnancy [6]. One of the six patients we operated on had all classic features of neurofibromatosis type 1 (NF1) with multiple neurofibromas. We removed two neurofibromas in that case which were causing localized pressure effects and pain. Excision of all lesions is not routinely advocated, owing to scar formation and localized recurrence of this tumor [6].

Basal cell carcinoma (BCC), being the most frequently mutating tumor in human beings, has a lifetime risk of 30% among the fair-skinned population in their sixth decade, with a double incidence in females. All cases had a predisposition for the face, with a propensity to the left side. No syndromic associations were found in any case. Histopathology reports of these patients showed a basaloid variant of BCC in all cases. A French review article establishes nodular variety as the most common histologic subtype though [2].

Squamous cell carcinoma (SCC) followed a fifty-fifty distribution rule in our patient population, whereby 50% of cases occurred in the upper limbs and another 50% in the face and scalp. Fifty percent of cases were right-sided, and another 50% were left-sided lesions. Cumulative sun exposure and immunosuppression are the two important risk factors for SCC. However, the majority of our patients had a positive history of the former risk factor [3].

Lesions with BCC and SCC were primarily managed by surgical excision in all patients, followed by radiotherapy and photodynamic therapy in some special cases. Other lesions, including spindle cell neoplasm, dermatofibroma, glomus tumor, and juvenile hyaline fibromatosis, were also present and were classified under miscellaneous lesions. They mostly involved the lower limbs (50%), upper limbs (25%), and scalp (25%).

Post-surgical complications cannot be taken for granted in cosmetic surgical procedures. The existing scientific literature shows that surgical site infections (SSI), local anesthetic systemic toxicity (LAST), electrolyte imbalance, fluid shifts, and hematologic abnormalities are the most often noted post-procedural complications [12]. Fever was the commonly observed post-surgical complication in 40% of patients in our department. Previously published articles on the occurrence of SSI in aesthetic surgery have described its prevalence as less than 1% in multiple procedures altogether [12]. A subset of individuals (10%) in our retrospective study also manifested SSIs, and 6.67% had graft or flap rejection. SSIs can cause sutural rupture and graft necrosis [13]. Transitory surgical edema and tissue collections are usually expected in plastic and reconstructive surgeries. However, persistent swellings may indicate hematoma formation. Wound site hematoma was also noted in 6.67% of operated cases. They can occur on the first postoperative day or many days after surgery [12].

The majority of SSTS are removed under local anesthesia, hence anxiety and post-operative pain are anticipated postoperative sequelae, especially in patients undergoing face and scalp surgery [13]. However, our patients received an adequate cover of analgesics before and after the procedures and this was not an observed complication. Hand-holding by nurses, listening to music, stress balls, and anxiolytic drugs are usually implied methods to decrease peri-operative restlessness and anxiety [13].

Our study, though the first of its kind on the topic of SSTS, has some major limitations. It is a single-center study, with a small sample size. The retrospective manner of its conduction and retrieval of limited data from patients’ data makes it difficult to generalize the results for a bigger population.

## Conclusions

Among the 60 patients included in this study, the majority were females in their fourth decade of life. The most common skin and soft tissue lesion noted was nevus followed by arteriovenous malformations, basal cell carcinoma, and squamous cell carcinoma, affecting the face and scalp in the predominant population. Clean surgical excision was the primary mode of treatment. The most common postoperative complications were fever and surgical site infections.

Although the number of patients approaching our department for the management of skin and soft tissue swellings has increased over the past few years, we deduce that there is a relative negligence of dermatologic swellings among men. Female patients are more concerned about their cosmetic appearance and sought surgical management. The apt surgical excision with fewer post-procedural complications at our center highlights the fact that cosmetic surgeries can be hassle-free and easily managed with standard measures of asepsis and appropriate surgical techniques. Histopathology of excised swellings is the most important trail in the path of management of these lesions.
